# Cyclodextrin-Containing Polymers: Versatile Platforms of Drug Delivery Materials

**DOI:** 10.1155/2012/262731

**Published:** 2012-02-02

**Authors:** Jeremy D. Heidel, Thomas Schluep

**Affiliations:** Calando Pharmaceuticals, Inc., 225 South Lake Avenue, Suite 300, Pasadena, CA 91101, USA

## Abstract

Nanoparticles are being widely explored as potential therapeutics for numerous applications in medicine and have been shown to significantly improve the circulation, biodistribution, efficacy, and safety profiles of multiple classes of drugs. One leading class of nanoparticles involves the use of linear, cyclodextrin-containing polymers (CDPs). As is discussed in this paper, CDPs can incorporate therapeutic payloads into nanoparticles via covalent attachment of prodrug/drug molecules to the polymer (the basis of the Cyclosert platform) or by noncovalent inclusion of cationic CDPs to anionic, nucleic acid payloads (the basis of the RONDEL platform). For each of these two approaches, we review the relevant molecular architecture and its rationale, discuss the physicochemical and biological properties of these nanoparticles, and detail the progress of leading drug candidates for each that have achieved clinical evaluation. Finally, we look ahead to potential future directions of investigation and product candidates based upon this technology.

## 1. Cyclosert: Rationale and Introduction

Ever since Paul Ehrlich introduced the concept of the “magic bullet”—that is, the combination of an agent conferring selectivity towards a disease-causing organism with a therapeutic agent—scientists have worked towards achieving this vision. One way to achieve selectivity towards certain disease states was to develop a prodrug that would be administered in its inactive and nontoxic form but would be metabolized to its active form once it reached the diseased organ. Prodrug approaches have been used by medicinal chemists to improve the absorption, distribution, metabolism, and excretion (ADME) of many small-molecule drugs. This approach was also important in increasing the selectivity of many small-molecule drugs, especially in the field of oncology. Examples such as irinotecan (a prodrug of the camptothecin analog, SN-38), capecitabine (a prodrug of 5-FU), and etoposide phosphate (a prodrug of etoposide) have shown clinical success and thereby demonstrated the value of this approach. This concept was further expanded through the development of macromolecular prodrugs. The rationale for using macromolecules as drug carriers is that they may be able to incorporate many more functional features than a relatively simple small molecule, therefore enabling them to perform complex functions at the right time and right place within a patient. A nanoparticle drug, one form of a large macromolecular drug, has a hydrodynamic diameter between ~10 and ~100 nm. Many types of nanoscaled drugs, such as antibody conjugates, polymer conjugates, and liposomal drugs, have been developed. The most important functional features of nanoparticle drugs are shown in Table 1.

**Figure 1:**
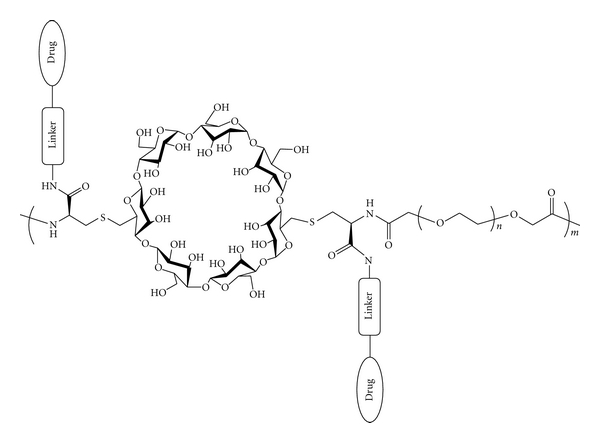
Structure of linear, cyclodextrin-based polymer (CDP) for small molecule delivery. The polymer consists of the cyclical sugar *β*-cyclodextrin that has been difunctionalized with the natural amino acid cysteine (CDDCys) and polyethylene glycol (PEG). Two small-molecule drugs per polymer repeat unit can be attached via various linker chemistries, resulting in a neutrally charged, highly water-soluble polymer conjugate.

Here, we discuss the preclinical and clinical development of a class of nanoparticles for the delivery of small-molecule drugs based on linear, cyclodextrin-based polymers (CDPs). CDPs contain alternating repeat units of *β*-cyclodextrin (CD) and polyethylene glycol (PEG) with two carboxylate groups per repeat unit for drug conjugation (Figure 1). Both components are commonly used in drug delivery applications. Cyclodextrins are cyclical sugar molecules with a hydrophilic exterior and hydrophobic cavity interior. High aqueous solubility and the ability to encapsulate hydrophobic moieties within their cavity through the formation of inclusion complexes enable cyclodextrins to enhance the solubility, stability, and bioavailability of hydrophobic small-molecule drugs [1]. PEG is often used in pharmaceutical applications to increase the solubility, stability and plasma half-life of drugs [2].

**Figure 2:**
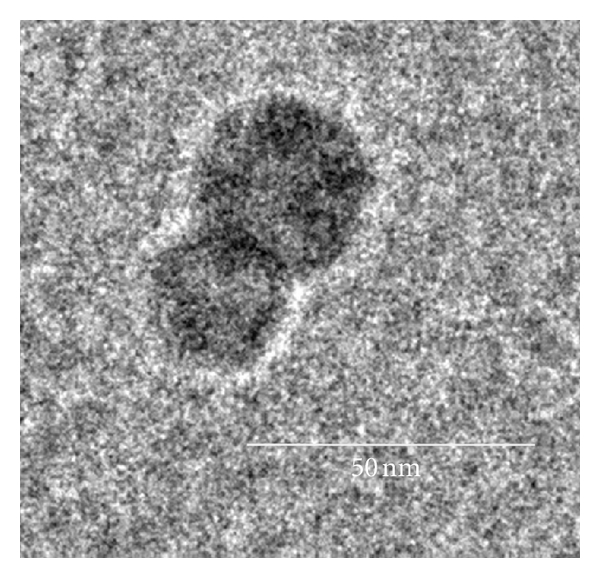
Transmission electron micrograph (TEM) of CRLX101 (from [8]).

In order to form the CDP polymers, a difunctionalized *β*-cyclodextrin is reacted with a difunctionalized PEG through condensation polymerization [3]. The resulting polymer is highly water soluble and neutrally charged when fully conjugated with drug through various linkers. This results in a high biocompatibility of the polymer, eliciting no observable side effects or immune responses at intravenous doses up to 240 mg/kg in mice [4]. A number of small-molecule drugs such as camptothecin [3], a natural alkaloid antineoplastic agent, tubulysin [5], a naturally occurring tetrapeptide with antineoplastic activity isolated from strains of myxobacteria, and methylprednisolone [6], an steroid anti-inflammatory drug, have been attached to CDP through various linkers (Table 2). One of the unique features of CDP is that the CD blocks form inclusion complexes with hydrophobic small-molecule drugs through both intra- and intermolecular interactions. Such interactions between adjacent polymer strands are essential for catalyzing the self-assembly of several CD-PEG polymer strands into highly reproducible nanoparticles (Figure 2). Parameters affecting the particle size are the type of drug, the polymer molecular weight, and the drug loading. Covalent attachment of a hydrophobic drug is required to initiate self-assembly, and release of drug from the polymer results in the disassembly into individual polymer strands of 8-9 nm, which have the potential to be cleared through the kidney [5–7].

 CDP-based nanoparticles are highly water soluble at concentrations >100 mg/mL, limited by the high viscosity of resulting solutions, increasing the solubility of hydrophobic drugs by more than 100-fold (Table 2). One attractive feature of nanoparticle prodrugs is their ability to protect small-molecule therapeutics from enzymatic and chemical degradation. This was impressively shown in the case of the camptothecin (CPT) drug, CRLX101 (formerly IT-101). The chemical structure of CPT includes an unstable lactone ring that is highly susceptible to spontaneous and reversible hydrolysis, which yields an inactive, but more water-soluble, carboxylate form that predominates at physiologic pH. To form CRLX101, CPT is derivatized at the 20-OH position with the natural amino acid glycine to form an ester linkage for covalent attachment to CD-PEG (Table 2). *In vitro* studies confirmed that this linker strategy successfully stabilizes the labile lactone ring of CPT in its closed, active form. Release of CPT from the nanoparticles was found to be mediated through both enzymatic and base-catalyzed hydrolyses of the ester bond, with observed half-lives of 59 and 41 hours in PBS and human plasma, respectively [3]. Release of methylprednisolone showed similar kinetics, with observed half-lives of 50 and 19 hours in PBS and human plasma, respectively [6]. These release kinetics are substantially slower than what is typically observed with nonnanoparticle ester prodrugs [9, 10] and this is most likely due to the displacement of water from within and reduced access of enzymes to the hydrophobic core of CDP nanoparticles. The disulfide linked ester conjugate was significantly more stable, with minimal release observed in PBS or human plasma over 72 hours [5].

The ability of any nanoparticle therapeutic to deliver the payload to the target cell and release it at the right time and location will be important for its performance. Release of the payload can be triggered by various mechanisms, depending on the linker chemistry. CDP polymers have been used in combination with ester linkages, such as glycine or triglycine, as well as disulfide linkers. While ester linkers are cleaved through pH-dependent and enzymatic hydrolysis, disulfide linkers are cleaved in response to a change in redox potential upon intracellular uptake of the nanoparticle. *In vitro* and *in vivo* studies showed that CDP nanoparticles are taken up by various cell types, including tumor cells and cells of the immune system [4, 7, 11]. Intracellular uptake and release are also directly correlated to the *in vitro* potency of the conjugate. In the case of CRLX101, the *in vitro* potency was found to be between one-half to one-tenth the potency of the unconjugated CPT in a 48-hour MTS assay [12]. In contrast, the *in vitro* potency for the disulfide-conjugated tubulysin nanoparticle was similar to that for the free drug in a 48-hour assay, consistent with a more rapid release after intracellular uptake [5]. The time dependence of *in vitro* potency was studied more extensively in the case of the ester-linked methylprednisolone nanoparticle, for which the potency of the nanoparticle at 5 days in a lymphocyte proliferation assay was higher than that of free drug [6]. In the same assay, the free drug was more potent at 3 days, consistent with the slow release of active drug from the nanoparticle over time.

## 2. Pharmacokinetics and Pharmacodynamics of Cyclosert-Based Nanoparticle Drugs

The ability of nanoparticles to dramatically change the pharmacokinetics (PK) and biodistribution of drugs on both a macroscopic level (i.e., whole organ) and a microscopic (i.e., cellular) level is key to achieving the desired improvements in pharmacodynamics (PD) and, ultimately, therapeutic index. Plasma PK after intravenous injection was extensively studied for CRLX101 by traditional HPLC assays in rats [13] and by micro-PET/CT in mice using ^64^Cu-labeled nanoparticles [7]. The nanoparticle PK is characterized by a low volume of distribution approximately equal to the total blood volume and long terminal half-life of 13 to 20 hours in mice and rats, respectively. This result indicates that the nanoparticles are able to avoid first-pass kidney clearance, which is commonly observed for drugs with hydrodynamic diameters below 10 nm [14]. This was in contrast to the PK of CPT alone, which showed a high volume of distribution and short terminal half-life of 1.3 hours.

After intravenous administration, CDP nanoparticles therefore form a circulating reservoir of active drug that is subsequently distributed to multiple organs. Consistently, tumor tissue showed high drug concentrations 24 to 48 hours after injection of nanoparticles. Other tissues with high drug concentrations were liver, spleen, and kidney, while most other organs showed low concentrations. A detailed study of multiorgan PK by PET/CT and histology revealed that CRLX101 nanoparticles were taking advantage of the unique tumor physiology characterized by a high density of abnormal blood vessels, high vascular permeability, and decreased rate of clearance due to a lack of lymphatic drainage, all of which act together to cause accumulation. This phenomenon has also been called the enhanced permeability and retention (EPR) effect [15]. In the same study, intact nanoparticles were found inside cancer cells distributed throughout the tumor tissue, forming an intracellular reservoir of active drug. This intracellular accumulation can also explain the finding that tumoral concentrations of CRLX101 and released CPT remained relatively constant for several days after intravenous injection, as opposed to the rapid decline (over several orders of magnitude in less than 24 hours) of irinotecan and its active metabolite, SN-38, in several preclinical lymphoma models [16]. These increased tumor concentrations also correlated with increased inhibition of topoisomerase I enzymatic activity at 48 hours after administration by CRLX101 compared to irinotecan.

Studies of the biodistribution and cellular uptake of fluorescently labeled CDP nanoparticles (NPs) in a syngeneic glioma model in C57BL/6 mice showed an additional mechanism of nanoparticle transport and distribution [11]. Irrespective of route of administration (intravenous versus intracranial), CDP-NPs were more efficiently taken up by tumor-associated macrophages (TAMs), macrophages, and microglia, than tumor cells. These TAMs not only internalized NPs by phagocytosis but also were able to migrate into the circulation after local intracranial CDP-NP injections. Additionally, NP-positive TAMs distributed to distant tumors within the CNS after local intracranial delivery. One unique characteristic of the fluorescently labeled CDP-NPs used for these studies was their slightly positive surface charge, while all of the other CDP-NPs discussed here had a slightly negative to neutral surface charge. Taken together, these observations indicate that CDP-NPs could be tuned to circulate as free NPs in plasma for prolonged periods of time and/or be taken up by immune cells such as TAMs and transported via cell migration. Both of these effects may occur at the same time and are not mutually exclusive. In addition to cancer, these findings of macrophage transport may have implications for the application of CDP-NPs in other indications, such as inflammatory diseases. It is conceivable that some of the enhanced *in vivo* activity of Cyclosert-methylprednisolone [6] was also due to immune cell-mediated transport.

One major benefit of these PK and PD improvements is a dramatic increase in therapeutic index for many small-molecule drugs. For example, CPT essentially has no therapeutic window and its development was abandoned due to excessive toxicity. CRLX101, in contrast, has shown to be highly active in multiple human subcutaneous and disseminated cancer models [16, 17]. In all cases studied, one treatment cycle of 3 weekly doses of CRLX101 resulted in significant antitumor activity that was superior to irinotecan or topotecan, two small-molecule analogs of CPT. In the case of tubulysin A (TubA), the increase in therapeutic index was even more impressive, showing a >100-fold increase in maximum tolerated dose (MTD). Whereas TubA at its MTD was completely inactive, CDP-TubA showed equal or superior efficacy compared with vinblastine and paclitaxel reference treatments with minimal observed toxicity [5]. While cancer is a natural indication for nanoparticle drugs, many other indications may be amenable to treatment with nanoparticle drugs. The common denominator in these diseases is the presence of inflammation resulting in similar physiological changes, such as neovascularization and high vascular permeability. Preclinical studies in models of rheumatoid arthritis showed that this approach can work for anti-inflammatory therapy and may be expanded to other disease indications [6].

## 3. CRLX101 Clinical Translation

Based on the preclinical activity of CRLX101, clinical development was initiated. This required a significant investment in process improvements and scale-up of nanoparticle manufacturing. Specific process challenges that had to be overcome were the control over the polymerization reaction, consistency of drug loading, and reproducible nanoparticle formation. In order to set appropriate specifications for key parameters potentially affecting the *in vivo* characteristics of the drug, a bracketing approach was chosen. Key nanoparticle specific parameters identified were polymer molecular weight (Mw) and drug loading, both of which are controllable by specific process control measures, as well as the particle size, which is a function of the two independent parameters (Table 3). A series of nanoparticle compounds bracketing each independent parameter were synthesized, their particle sizes determined, and pharmacokinetics and pharmacodynamics evaluated *in vivo*. Results of these studies were then used to set upper and lower specification limits for both independent and dependent variables.

A phase I study of CRLX101 in patients with refractory solid tumors was initiated. The primary objectives of this first-in-man study were to determine the safety, pharmacokinetics, dose-limiting toxicities, and MTD, as well as the recommended dose and dosing schedule for future studies. Secondary objectives of the study included the assessment of potential biomarkers, an estimation of clinical activity by RECIST, and an estimation of progression-free survival in patients receiving multiple cycles of CRLX101 monotherapy. Interim results of that study are available [18].

Patients with refractory solid tumors received CRLX101 using either 3 weekly (Qwkx3) or every other week (Qow) infusions every 28 days. CRLX101 was administered at 6, 12, or 18 mg/m^2^ Qwkx3 and 12 or 15 mg/m^2^ Qow. The occurrence of adverse events during the first cycle was used to assess the toxicokinetics. As of the interim analysis, eighteen patients had been enrolled; of these, 12 patients received CRLX101 Qwkx3 and 6 Qow. Consistent with preclinical results, CRLX101 showed a long elimination half-life of 31.8 and 43.8 hours for polymer-bound and free CPT, respectively. Volume of distribution of the polymer conjugate was 4.2 ± 1.1 liters, indicating that CRLX101 is initially primarily retained in the vasculature. An analysis of toxicokinetics in patients that received CRLX-101 either on the Qwkx3 or Qow schedule showed that tolerability was improved on the Qow regimen while maintaining similar per-cycle drug exposures. Hematologic toxicity was dose limiting at 18 mg/m^2^ on the weekly schedule. The authors concluded that CRLX101 given intravenously appeared safe when administered between 18 and 30 mg/m^2^/month in both Qwkx3 and Qow regimens; however, the Qow schedule was better tolerated. More recently [19], data from additional patients dosed on the Qow regimen highlight observations of stable disease in advanced non-small-cell lung carcinoma (NSCLC) patients. Specifically, the interim data showed that 70% of the NSCLC patients achieved stable disease of greater than or equal to 3 months, and 20% of them achieved stable disease of greater than or equal to 6 months. Accrual of this phase I study has since been completed, and a randomized phase 2 study of CRLX101 in patients with advanced NSCLC has been initiated. Results from these upcoming studies will be critical for establishing the potential of CRLX101 as a new oncology agent.

## 4. RONDEL: Introduction and Rationale

**Figure 3:**
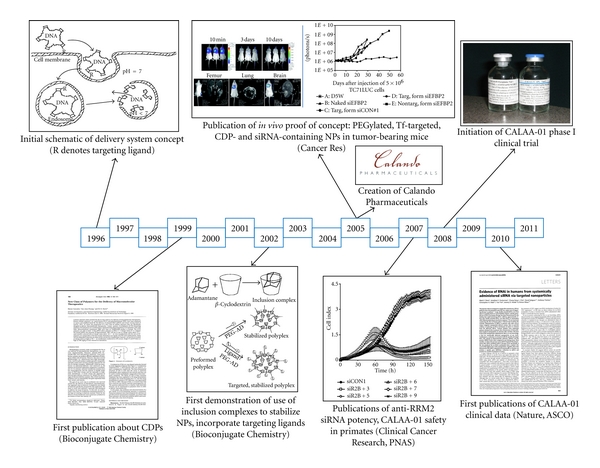
Timeline of the development of cyclodextrin-containing polymers (CDPs) for nucleic acid delivery.

The development of linear cyclodextrin-containing polymers (CDPs) for nucleic acid delivery traces back to the mid-1990s in the laboratory of Dr. Mark Davis at Caltech (Figure 3). In order to function as delivery agents for polyanionic nucleic acids, of which DNA oligonucleotides and plasmid DNA (pDNA) were most prevalent at that time, cationic polymers were conceived by Dr. Davis as those that would contain several key attributes: (i) assemble with nucleic acids to yield small (~100 nm or below in diameter) colloidal particles, (ii) could be easily modified with a stabilizing agent (e.g., poly(ethylene glycol) (PEG)) and a targeting ligand to facilitate *in vivo* stability and engagement of cell surface receptors on target cells and promote endocytosis, and (iii) respond to vesicular acidification as a trigger to escape the endosome and trigger particle disassembly, thereby releasing the nucleic acid payload within the cytoplasm. Cyclic oligomers of glucose, cyclodextrins were selected as the foundation of these polymers because of their known low toxicity, lack of immunogenicity, and ability to form noncovalent guest-host inclusion complexes with hydrophobic small molecules; the first description of the synthesis of a cationic CDP and characterization of the nanoparticles it formed with pDNA, including their *in vitro* transfection efficiency, was published in 1999 [20]. To overcome the salt-induced aggregation of CDP/pDNA nanoparticles in physiological media, chemistry was developed to conjugate a neutral stabilizing polymer, PEG, to a hydrophobic small molecule, adamantane (AD), which forms strong inclusion complexes with *β*-cyclodextrin. In this manner, nanoparticles could be noncovalently stabilized, and this approach was extended to allow incorporation of targeting ligands via preparation of AD-PEG-ligand conjugates [21, 22]. Utilizing a small interfering RNA (siRNA) targeting the EWS/Fli1 fusion oncogene and the human transferrin protein as a targeting ligand, the first *in vivo* proof-of-concept experiments were performed shortly thereafter in a disseminated murine model of Ewing's sarcoma [23]. The significant antitumor effect demonstrated in this work motivated the creation of a company, Calando Pharmaceuticals, to further advance this delivery platform (RONDEL) towards therapeutic candidates suitable for clinical evaluation in human cancer patients. The first such candidate, termed CALAA-01, contained an siRNA targeting the M2 subunit of ribonucleotide reductase (RRM2), a protein involved in DNA replication whose function is required to complete cell division. Upon identification of the optimal anti-RRM2 siRNA sequence [24] and evaluation of the *in vivo* nanoparticle performance [25], an IND application was submitted to the Food and Drug Administration (FDA) and Calando received approval to initiate a phase I trial of CALAA-01 in patients with solid tumors in 2008. In 2010, encouraging interim clinical data from this study was published [26, 27] which revealed, in addition to a promising safety profile and multiple dose escalations, the first evidence of the RNA interference (RNAi) mechanism of action in humans and the first dose-dependent tumor accumulation in humans of nanoparticles of any kind upon systemic administration.

In this paper, we describe the development of each of the components of this nucleic acid delivery system. We review the assembly of these nanoparticles, including their physicochemical properties and *in vivo* performance. The development of the CALAA-01 drug product is then discussed, including selection of the gene target and siRNA sequence optimization, safety and efficacy evaluations in animals, and manufacturing/scale-up of the components. The clinical findings of CALAA-01 are then discussed, including characterization of safety parameters (pharmacokinetics (PK), complement activation, cytokine levels, serum chemistry, complete blood counts (CBCs), and adverse events), and efficacy and a discussion of exploratory objectives. Finally, we conclude with a survey of additional explorations conducted with this delivery platform with an eye towards next-generation therapeutic candidates.

## 5. RONDEL Components

Fully formulated nanoparticles made with the RONDEL (RNAi/Oligonucleotide Nanoparticle Delivery) system, such as the CALAA-01 drug product developed by Calando Pharmaceuticals currently in clinical evaluation, contain a total of four (4) components described below.

**Figure 4:**
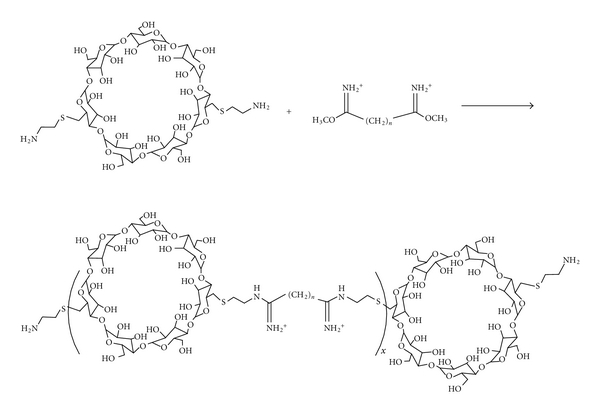
Polymerization scheme to yield amine-terminated CDP (from [32]).

**Figure 5:**
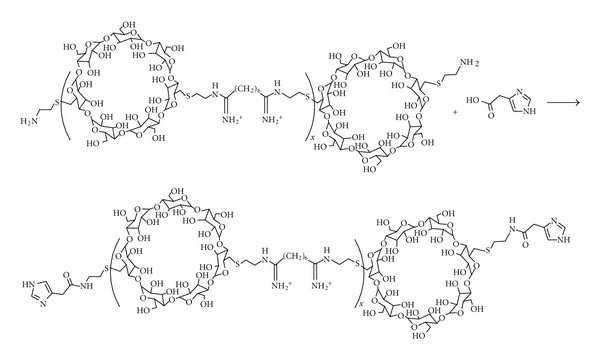
Polymer modification scheme to incorporate imidazole derivative within CDP.

**Figure 6:**
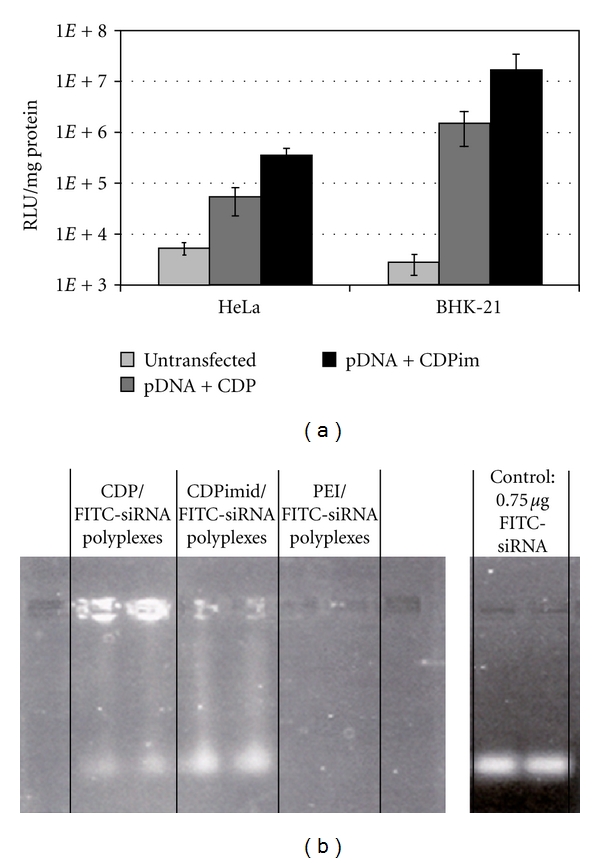
Effect of imidazole incorporation within CDP upon gene delivery efficiency and intracellular siRNA release. (a) Incorporation of an imidazole derivative within CDP (CDPim) leads to a significant increase in transgene (luciferase) expression levels in transfected HeLa and BHK-21 cells (from [33]). (b) Imidazole incorporation (CDPimid) yields a significant (~4x) increase in the fraction of intracellular siRNA that is released from the polymer and able to migrate through an agarose gel when electrophoresed (from [34]).

The three primary cyclodextrins (CDs)—*α*, *β*, and *γ*—are cyclic oligomers comprised of 6, 7, and 8 glucose moieties, respectively. Functionalization and polymerization efforts were conducted with these cyclodextrin species as part of several studies to assess structure-activity relationships (SARs) of cationic polymers varying in properties such as carbohydrate size, carbohydrate distance from charge centers, and charge center type [28–31]. In general, the cyclodextrins were difunctionalized and reacted with a difunctional comonomer to yield linear, AB-type copolymers (Figure 4). A number of trends emerged from these SAR studies (Table 4) which led to the identification of a preferred structure for the CD-containing polymer (CDP) which was the focus of further development (Figure 5). Designated as “*β*CDP6,” “CDPim,” or “CAL101” in various publications (hereafter referred to as CAL101), this polymer is made by copolymerization of *β*-CD diamine and dimethylsuberimidate (which imparts two amidine charge centers separated by six methylene units), and its termini are modified to contain an imidazole derivative. This modification has been shown to facilitate enhanced transgene expression from a plasmid DNA (pDNA) payload and to significantly release intracellular release of siRNA (Figure 6). Nanoparticles made with CAL101 and pDNA yielded significant gene delivery in transfected cultured cells, comparable to that of leading commercially available transfection reagents, with low cytotoxicity. Despite this *in vitro* potency, these charged colloidal CAL101/nucleic acid nanoparticles rapidly aggregate in physiological medium, rendering them unfit for *in vivo* application; this phenomenon motivated investigation into incorporation of a stabilizing agent.

The objectives of addition of a stabilizing agent to CAL101-containing nanoparticles are to minimize self-self (aggregation) and self-nonself (e.g., protein binding) interactions in an animal or human subjects receiving a systemic administration of these nanoparticles. For cancer treatment in particular, it is known that passive targeting of nanoparticles to tumors can occur through a prolonged circulation time which enables extravasation through fenestrated tumor neovasculature (enhanced permeation and retention (EPR) effect). Thus, in order to direct the biodistribution of CAL101/nucleic acid nanoparticles such that tumor uptake is maximized (and the potential for off-target deposition and toxicities are minimized), efforts to incorporate a neutral polymer, PEG, to stabilize these nanoparticles were undertaken.

**Figure 7:**
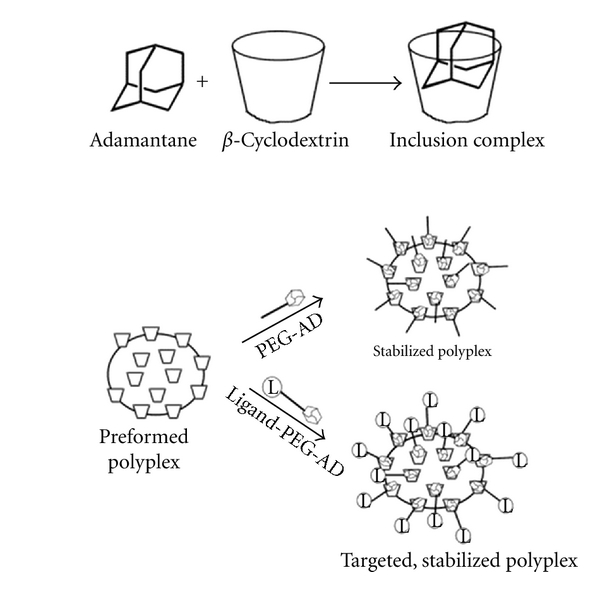
Formation of inclusion complexes between adamantane (AD) and *β*-cyclodextrin allows straightforward, noncovalent incorporation of stabilizing (via PEG-AD conjugates) and/or targeting (via ligand-PEG-AD conjugates) components to a polymer-nucleic acid nanoparticles (polyplex) (Figure from [21]).

While PEGylation of cationic polymer-based nanoparticles to extend circulation times and prevent aggregation was widely performed, it typically required covalent attachment of PEG at the same polymer functional sites required for nucleic acid binding. This tradeoff is undesirable, and it was overcome in this case due to exploitation of the *β*-CD moiety within CAL101 (Figure 7). Forming strong noncovalent inclusion complexes with *β*-CD (association constant of ~ 10^4^-10^5^ M^−1^), adamantane (AD) was conjugated to one terminus of a linear PEG (AD-PEG) and added to CAL101 either before (pre-PEGylation) or after (post-PEGylation) CAL101 had been combined with the nucleic acid of interest. In this manner, simple physical mixing of these components was sufficient to achieve sufficient interaction and incorporation of AD-PEG into the nanoparticles. A minimum PEG length of 5 kDa was shown to be required to prevent salt-induced aggregation of these nanoparticles [21], and thermodynamic analysis suggests that length-dependent interactions among PEG chains on the surface of nanoparticles contribute significantly to the effective stabilization [36]. This AD-PEG_5000_ conjugate was the focus of future development work for this RONDEL delivery platform as well as clinical translation of the CALAA-01 therapeutic candidate.

**Figure 8:**
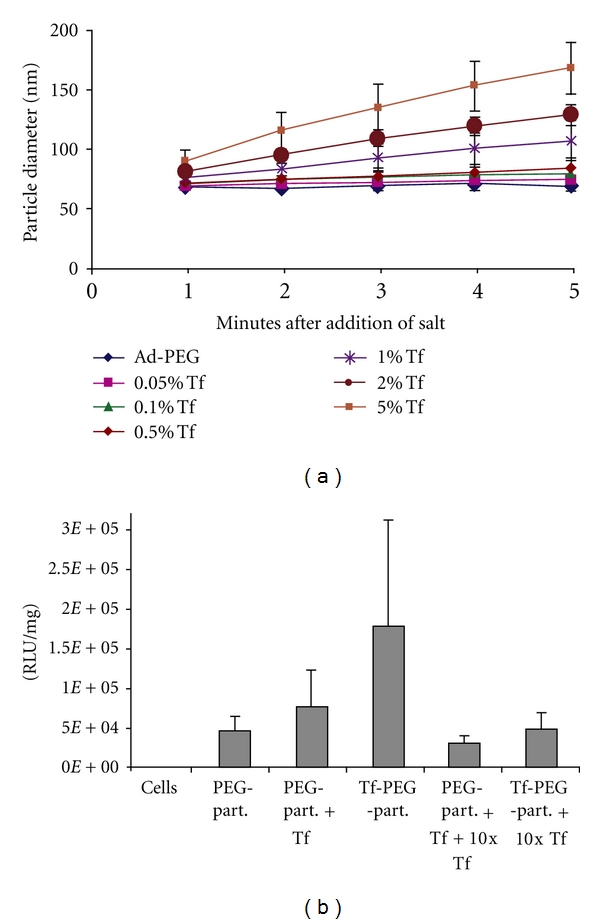
Effect of AD-PEG-Tf incorporation on nanoparticle size, salt stability, and transgene efficiency. (a) Dynamic light scattering (DLS) measurements of nanoparticle size as a function of time after the addition of salt (phosphate-buffered saline) help to define an optimal formulation window above which excessive AD-PEG-Tf leads to salt-induced nanoparticles aggregation. Nanoparticles were prepared containing 0% (Ad-PEG) or the indicated mol% of AD-PEG-Tf (percentage of total cyclodextrins by mole, with the remaining balance to 100% comprised of AD-PEG). (b) When plasmid-containing nanoparticles are exposed to cultured cells, inclusion of AD-PEG-Tf in the formulation increases transgene expression in a manner that can be reversed by addition of soluble Tf as a competitor, suggesting that TfR-mediated endocytosis plays a role in nanoparticle uptake and/or intracellular trafficking. Treatments included cell alone (cells), non-AD-PEG-Tf-containing nanoparticles (PEG-part.), non-AD-PEG-Tf-containing nanoparticles plus 0.05 mol% free soluble Tf (PEG-part. + Tf), AD-PEG-Tf-containing nanoparticles (Tf-PEG-part.), non-AD-PEG-Tf-containing nanoparticles plus 10 equivalents of free soluble Tf (PEG-part. + Tf + 10x  Tf), or AD-PEG-Tf-containing nanoparticles plus 10 equivalents of free soluble Tf (Tf-PEG-part. + 10x  Tf) (from [22]).

Having included CAL101 as a condensing agent to induce nanoparticle formation and AD-PEG as a stabilizing agent to render these nanoparticles suitable for *in vivo* application, a third component was investigated which would facilitate cellular internalization of nanoparticles. Typical candidates for such an agent in nanoparticle formulations are ligands (in the form of peptides, proteins/antibodies, aptamers, or small molecules) whose cognate receptor is expressed on the surface of target cells either exclusively or to a much greater extent than on other (nontarget) cells. For application of these nanoparticles to cancer, the transferrin receptor (TfR) was selected [22] as a target owing to its significant overexpression on a variety of cancer cell types [37]; indeed, TfR is a well-studied surface protein for targeting of cancer therapeutics [38, 39]. The aforementioned AD/*β*-CD inclusion complex phenomenon was exploited to incorporate the human transferrin (Tf) protein to these nanoparticles. Specifically, Tf was conjugated to the distal end of a functionalized AD-PEG_5000_ to yield an AD-PEG_5000_-Tf species which could also contribute to nanoparticles via physical mixing with the other components. Owing to the significant size (~80 kDa) and net anionic charge of Tf, the range of stoichiometries which would retain desired nanoparticles size and stability while yielding a biological effect was established (Figure 8). As is discussed below and has been reviewed previously [40], the presence of AD-PEG-Tf within these nanoparticles does not significantly alter their overall biodistribution but appears to enhance activity *in vivo*, presumably through enhanced internalization by cancer cells.

The final component of the nanoparticles, the siRNA, is typically a canonical siRNA (two 21-nucleotide strands sharing 19 nt of Watson-Crick complementarity with 2-nt, 3′ overhangs) although successful formulation with alternative RNAi constructs has been observed. Because protection from serum nucleases is afforded by formulation within CAL101-containing nanoparticles, replacement of native phosphodiester linkages with phosphorothioates (which impart nuclease resistance) was not performed. In addition, because preclinical investigation did not reveal evidence of strong immunogenicity at therapeutically relevant dose levels (as discussed below), siRNA modifications that may reduce cytokine activation via Toll-like receptor (TLR) interaction, such as 2′-OMe and 2′-F, were not imposed. As a result, the siRNA species investigated within these nanoparticles as described in this paper are truly native/unmodified species whose degradation products are naturally occurring and require no special chemistries to synthesize.

The modular nature of these siRNA-containing nanoparticles affords flexibility with respect to the means and order of assembly by which they are formulated. Two distinct orders of assembly (“post-PEGylation” versus “pre-PEGylation”) can be employed. For post-PEGylation, CAL101 is combined with siRNA to form polyplexes to which PEG-containing species (i.e., AD-PEG and AD-PEG-Tf) are subsequently added. By contrast, a pre-PEGylation approach involves combining all three delivery system components together to yield a mixture which is then added to siRNA. Both strategies can provide nanoparticles <100 nm in diameter that demonstrate resistance to salt-induced aggregation. Because it involves a single mixing step to create nanoparticles at the time of use, the pre-PEGylation strategy was employed for the nanoparticle investigations described in the remainder of this paper. In addition, because of nearly instantaneous nanoparticle formation with this approach and reproducibility with respect to physicochemical properties, subsequent investigation of these siRNA-containing nanoparticles involved formulation at the time of use—that is, rather than prepare a large quantity of nanoparticles in advance and store them, separate preparations of (i) combined delivery components and (ii) siRNA were provided which were mixed to yielded nanoparticles on the day of administration. This approach eliminated the need to demonstrate long-term nanoparticle storage stability and, owing to a single mixing step, permitted a facile preparation protocol to which it was easy for personnel at animal facilities and hospital/clinic pharmacies to adhere.

## 6. RONDEL Proof of Concept in Tumor-Bearing Mice: Expanded Nanoparticle Characterization

**Figure 9:**
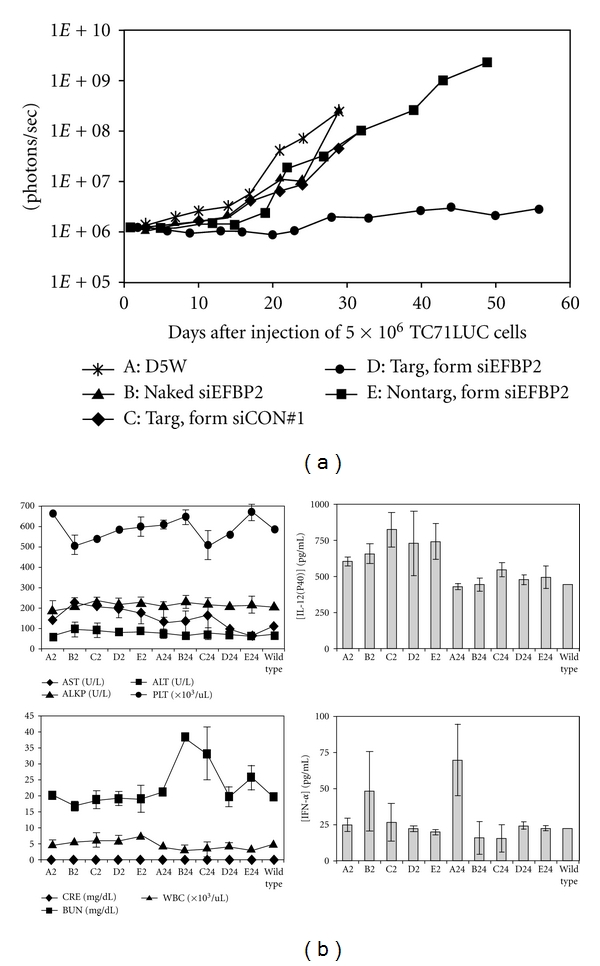
RONDEL-based nanoparticles containing siRNA against EWS/Fli-1 were well tolerated by mice and efficacious in a disseminated murine model of Ewing's sarcoma. (a) When administered twice weekly for four weeks, only nanoparticles containing AD-PEG-Tf and anti-EWS/Fli-1 siRNA (D) were effective in reducing tumor burden, as measured as integrated bioluminescence. Treatment group definitions: (A): vehicle control (D5W, 5 wt% dextrose in water), (B): anti-EWS-Fli1 siRNA without any other nanoparticle components (Naked siEFBP2), (C): nontargeting siRNA within Tf-targeted nanoparticles (Targ. form siCON#1), (D): anti-EWS-Fli1 siRNA within Tf-targeted nanoparticles (Targ. form siEFBP2), (E): anti-EWS-Fli1 siRNA within non-Tf-targeted nanoparticles (Nontarg. form siEFBP2). (b) When administered once to immunocompetent mice, these nanoparticles were well tolerated with respect to liver enzymes (aspartate transaminase (AST), alanine transaminase (ALT), alkaline phosphatase (ALKP), platelets (PLTs), indicators of kidney function (blood urea nitrogen (BUN) and creatinine (CRE)), and cytokine response (IL-12(p40) and IFN-*α*). Wild type denotes untreated mice; all other results are indicated by treatment group letter (A–E), as defined above, and the time point (2 or 24, in hours) at which blood was sampled (from [23]).

Having developed small-scale synthetic procedures for the three aforementioned components of the delivery system (CAL101, AD-PEG, and AD-PEG-Tf), an appropriate *in vivo* model was sought for a proof-of-concept investigation of the ability of this system to deliver siRNA to tumor cells in mice. In collaboration with Dr. Timothy Triche and colleagues at Children's Hospital Los Angeles, a disseminated murine model of Ewing's family of tumors (EFT)—mesenchymal malignancies that arise in bone or soft tissue or present as primitive neuroendocrine tumors and typically affect teenagers—was identified and selected. The vast majority (85%) of EFT patients have a unique chromosomal translocation that results in the creation of a chimeric EWS-Fli1 fusion that serves as an oncogenic transcription factor. Accordingly, siRNA species targeted specifically to the region of fusion had been described [32] which could induce apoptosis of EFT cells. A potent published anti-EWS-Fli1 siRNA was utilized within Tf-targeted nanoparticles to investigate the effect of treatment on cumulative tumor burden in mice. To create a disseminated EFT model in mice for which tumor burden could be readily measured, systemic (tail vein) injections were made of EFT cells which constitutively expressed firefly luciferase; this allowed the use of whole-animal bioluminescence imaging to quantify tumor burden. Employing a twice-weekly dosing regimen for four weeks, a statistically significant reduction in tumor burden was observed only for those nanoparticles which contained (i) the anti-EWS-Fli1 siRNA and (ii) the Tf targeting ligand (Figure 9(a)). Importantly, this was achieved in the absence of strong indications of toxicity or immunogenicity in these animals (Figure 9(b)). Together, these findings suggested a strong potential for continued development of this platform of siRNA-containing nanoparticles as anticancer therapeutics.

Even as these proof-of-concept results were obtained and Calando Pharmaceuticals was established (in 2005) to continue development of therapeutic candidates, research into the fundamental nature and behavior of these siRNA-containing nanoparticles continued in the laboratory of Mark Davis at Caltech. Two important publications in 2007 provided a more comprehensive physicochemical and *in vitro* biological characterization of these nanoparticles [36] and examined their biodistribution and pharmacokinetics in mice [41], respectively. A summary of the characterization findings is provided in Table 5. Notably, a combination of multiple experimental methods, including multiangle laser light scattering (MALS), allowed determination of nanoparticle stoichiometry—a 70 nm nanoparticle contains an average of ~10,000 CAL101 molecules, ~4000 AD-PEG molecules, ~100 AD-PEG-Tf molecules, and ~2,000 siRNA molecules. In addition, it was shown that the net ratio of positive (from CAL101) to negative (from siRNA) charges in the nanoparticles is ~1, implying that all additional CAL101 in the formulation remains as “free” (non-nanoparticle-contained). Since it is free components that are likely responsible for toxicity seen as high nanoparticles doses in animals (as discussed below), this finding suggests a strategy for potentially improving the therapeutic window of these formulations via removal/reduction in the levels of free components. To further examine the *in vivo* properties of these nanoparticles, positron emission tomography (PET)/computerized tomography (CT) was employed to monitor whole-body biodistribution kinetics and tumor localization of nanoparticles while concurrently using bioluminescence imaging to measure the ability of the nanoparticles (which contained antiluciferase siRNA) to downregulate their target in luciferase-expressing tumors. Comparing Tf-containing (targeted) versus non-Tf-containing (nontargeted) analogue formulations, it was revealed that both formulations exhibited similar biodistribution and tumor localization as measured by PET; however, compartmental modeling showed that a primary advantage of targeted nanoparticles was associated with processes involved in cellular uptake by tumor cells, rather than overall tumor accumulation. Thus, as has been discussed before [40], the term “internalization ligand” might well replace “targeting ligand” to describe the role of Tf in these nanoparticles. In addition, as had been shown in the EFT work described above, only targeted nanoparticles in this study were able to achieve a significant reduction in the expression level of the gene target in tumor cells.

## 7. RONDEL Translation: Calando Pharmaceuticals and CALAA-01

Founded in 2005, Calando Pharmaceuticals' mission is to develop drug delivery solutions to unlock the promise of RNAi therapeutics. The company designated the nanoparticle delivery system comprised of the cyclodextrin-containing polycation and adamantane-based stabilization and internalization components as its RONDEL platform. In addition to focusing on further advancing the analytical methodologies and manufacturing capabilities (as discussed below) for these components, Calando made substantial effort in the early days to identify an initial cancer gene target suitable for eventual clinical application and to optimize an siRNA to downregulate that target. Calando selected the M2 subunit of ribonucleotide reductase (RRM2), an established anticancer target which catalyzes a rate-limiting step in the production of 2′-deoxyribonucleoside 5′-triphosphates which are necessary for DNA replication. Just as TfR overexpression across a variety of cancer types opened the possibility of RONDEL-based nanoparticles to achieve uptake in many different classes of tumors, the demonstrated sensitivity of many cell types to RRM2 inhibition maintained the potential generality of anti-RRM2 siRNA-containing nanoparticles to treat multiple types of cancers. A combination of *in silico* and *in vitro* screening of many siRNA candidates led to identification of a lead sequence (named “C05C”; also referred to as “siRRM2B + 5”) which was shown to be a potent downregulator of RRM2 in cancer cells of various types and species and induced a concomitant antiproliferative effect in those cells [24].

Having defined the four components of Calando's putative lead candidate formulation (CAL101, AD-PEG_5000_, AD-PEG_5000_-Tf, and C05C), named “CALAA-01,” campaigns to scale up the manufacturing of each component were made while simultaneously expanding and improving the analytical methods employed to characterize each of them. The manufacturers of the lots of components used for IND-enabling toxicology studies and initial clinical material are listed in Table 6. Improvements in scale of up to three orders of magnitude were achieved for these molecules, and, as is customary for such projects, several challenges were identified and overcome during development. For example, in the case of CAL101, a previously unidentified impurity created in the initial *β*-CD functionalization step was observed that could be carried through subsequent steps; methodologies for quantifying and removing this species were developed and employed. Ultimately, sufficient quantities of all components were obtained that satisfied all acceptance criteria and were employed for subsequent testing.

Preclinical safety and efficacy testing of CALAA-01 were performed across a number of species and tumor types, respectively. A dose-range-finding study in non human primates [25] provided an early glimpse of the safety profile at each of three different dose levels (3, 9, and 27 mg/kg with respect to C05C). Several key findings were made in this study, including (i) the nature of toxicity at the highest dose levels (elevations in blood urea nitrogen and creatinine, as well as mild transient elevations in transaminase levels, indicative of kidney and liver effects, resp.), (ii) induction of mild levels of antinanoparticle (specifically, anti-Tf) antibodies that did not affect pharmacokinetics, (iii) elevation in IL-6 at the highest (27 mg/kg) dose level, and (iv) identification of relatively fast nanoparticle clearance from circulation (*t*_1/2_ < 30 min). Importantly, the overall safety profile indicated good tolerability at the 3 and 9 mg/kg dose levels, in the range for which antitumor effects had been observed. Additional (unpublished), more comprehensive toxicology and safety-pharmacology studies were performed in four species (mouse, rat, dog, and nonhuman primate) which provided a foundation for an initial clinical dose level and anticipated toxicities. In terms of efficacy, a twice-weekly dosing regimen of CALAA-01 yielded a significant reduction in tumor burden in mouse subcutaneous tumor models, including liver and melanoma [42], at dose levels in the range of 2.5–10 mg/kg.

**Figure 10:**
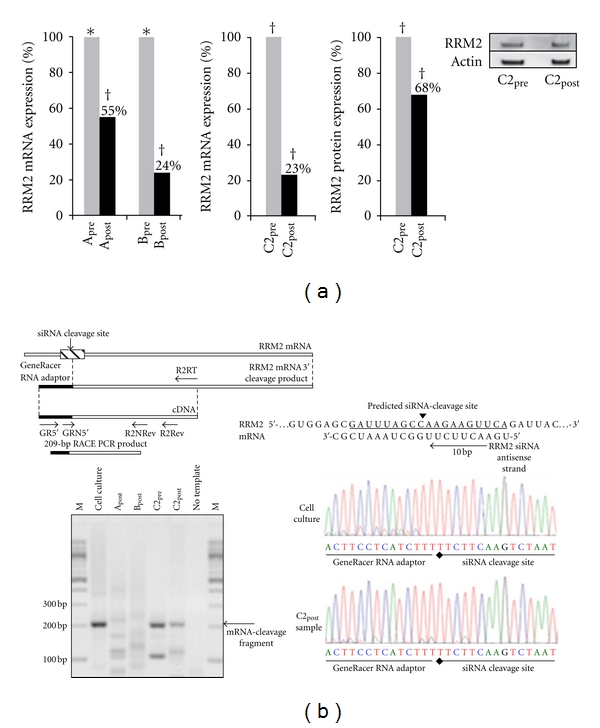
Interim data from a first-in-man, phase I clinical evaluation of CALAA-01 reveals RRM2 down regulation via an RNAi mechanism of action. (a) Measurements of RRM2 mRNA or protein levels in tumor biopsies from three patients (A, B, and C2) obtained before or after CALAA-01 treatment reveal significant reductions in target expression levels. (b) 5′-RLM-RACE analysis of RNA from one patient (C2) reveals evidence of the precise RRM2 mRNA cleavage product expected from RNAi-mediated down-regulation from the C05C siRNA contained within CALAA-01. This was the first such evidence of the RNAi mechanism of action in humans of any kind (from [26]).

CALAA-01 preclinical evaluation culminated in the submission of an Investigational New Drug (IND) application which received approval in April 2008. Shortly thereafter, a first-in-humans phase I investigation of CALAA-01 in patients having solid tumors was initiated. Patients who were refractory to standard-of-care treatment received four twice-weekly infusions (days 1, 3, 8, and 10) during a 21-day cycle over which numerous safety evaluations were made. CT assessments of tumor burden were performed, and PET assessment of tumor metabolism was also made. For volunteers willing to provide biopsies, assessments of RRM2 levels and investigation of the RNAi mechanism of action were also performed. At the time of this writing, a phase Ib study remains open, but interim clinical data have been published [26, 27]. Several dose level escalations spanning an order of magnitude (3, 9, 18, 24, and 30 mg/m^2^) have been tolerated, and key observations of RRM2 downregulation have been made in multiple patients. Pharmacokinetics indicate relatively fast clearance, consistent with preclinical findings, and some transient elevations in cytokines (IL-6, IL-10, and TNF-*α*) were seen. Importantly, the first evidence of the RNAi mechanism in humans (for any siRNA) and the first evidence of dose-dependent tumor accumulation of nanoparticles administered systemically in humans (for any nanoparticles) have been observed in this study (Figure 10). Taken together, these early indications of safety and efficacy suggest potential for CALAA-01 and the RONDEL platform for continued clinical investigation.

## 8. RONDEL and CALAA-01: Future Directions

Supported by over a decade of research and development, there are many ongoing and future directions for CALAA-01 and the RONDEL delivery platform. Certainly completion of the CALAA-01 phase I clinical trial, including establishment of a maximum tolerated dose (MTD) and recommended dose level for subsequent trials, is a near-term priority. Thorough evaluation of all of the safety and preliminary efficacy indications from this study will greatly inform the design of a phase II investigation of CALAA-01. Beyond CALAA-01, investigation of additional therapeutic candidates employing the RONDEL system, such as those targeting hypoxia-inducible factor-2*α* (HIF-2*α*), has been undertaken. The relatively fast clearance of these nanoparticles that has been observed, as has been described above, suggests that strategies to prolong circulation in an effort to enhance tumor accumulation may warrant investigation. The transient elevations in some cytokine levels seen in interim CALAA-01 clinical data imply that exploration of chemical modifications to the siRNA payload may yield nucleic acids that enhance the nanoparticles therapeutic index. With encouraging interim clinical data in hand, avenues for continued development and improvement of nanoparticles identified, and the emergence of alternative siRNA-containing nanoparticles in the clinic from which all in this field will learn, the future for siRNA-containing nanoparticles based on cyclodextrin-containing polycations appears bright.

## 9. Conclusions

CDP-based nanoparticles have made the transition from the laboratory to the clinic within the last several years. Two technology platforms have been developed, Cyclosert for small molecule delivery and RONDEL for nucleic acid delivery. Both programs have produced a clinical candidate for oncology, CRLX101 (formerly IT-101), a camptothecin analog, and CALAA-01, an siRNA therapeutic targeting RRM2. While clinical development is still in the early phases, proof of concept was achieved for both technologies. Clinical development is ongoing and it will be interesting to see what patient benefits these innovative drugs can provide.

## Figures and Tables

**Table 1:** Key nanoparticle characteristics and their effect on *in vivo* functionality.

Nanoparticle characteristics	Function
Diameter between 10 and 100 nm	Control over pharmacokinetics and biodistribution
Surface properties (charge, hydrophilicity)	Solubility, protection from aggregation, and interaction with cells and proteins
Core properties	Protection of payload from chemical and enzymatic inactivation, control of release kinetics
Linker chemistry	Protection of drug from chemical or enzymatic inactivation, control of release kinetics
Targeting ligands	Control of cell surface binding and intracellular uptake

**Table 2:** Linkers and drugs evaluated with the CDP nanoparticle system. The cleavage position is indicated with an arrow.

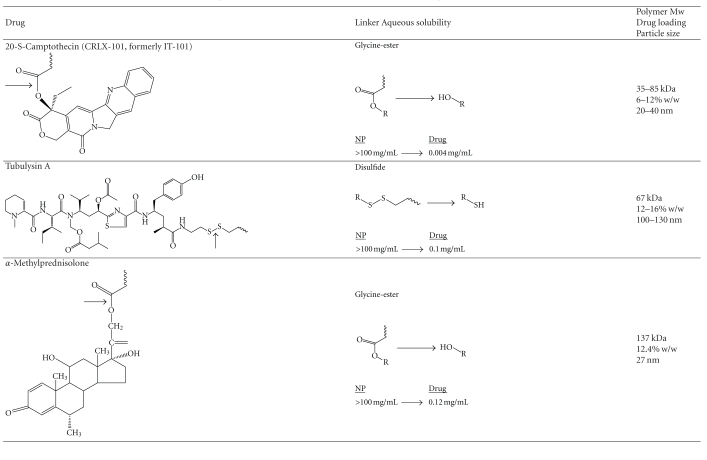

**Table 3:** Nanoparticle-specific independent variables, process control measures, and dependent variables used in setting specifications for Cyclosert drugs.

Independent variable	Process control measures	Dependent variable
Polymer molecular weight and polydispersity	Real-time viscosity determination during polymerization	Particle size
Drug loading	Stoichiometry of coupling reaction	Particle size, release kinetics

**Table 4:** Parameters and result summaries for early investigations of polymer structure-activity relationships (SARs).

Parameter	Variants tested	Results/trends	Reference(s)
Carbohydrate size	(i) Trehalose (ii) *α*-CD (iii) *β*-CD (iv) *γ*-CD (v) (hexane)	(i) The absence of a carbohydrate produces high toxicity and reduces water solubility (ii) CD-containing species had comparable properties	[29]

Carbohydrate distance from charge centers (spacer length) and hydrophilicity of the spacer	(i) 4, 5, 6, 7, and 8 versus 10 methylene units (ii) alkyl versus alkoxy spacers	(i) Transfection efficiency is dependent on distance from charge centers, with up to 20-fold difference among *β*-CD-containing polymers	[28, 29, 31]
		(ii) As the charge center is further removed from the carbohydrate unit, the toxicity is increased	
		(iii) Optimum transfection is achieved with a spacer length of 6 methylene units	
		(iv) Increasing hydrophilicity of the spacer (alkoxy versus alkyl) provides for lower toxicity	

Cyclodextrin functionalization	(i) 6^A^, 6^D^-Dideoxy-6^A^, 6^D^-Diamino-*β*-CD (ii) 3^A^, 3^D^-Dideoxy-3^A^, 3^D^-Diamino-*β*-CD (iii) 3^A^, 3^B^-Dideoxy-3^A^, 3^B^-Diamino-*γ*-CD	(i) The structure of diaminated CD monomers was found to influence both the molecular weight and polydispersity of polycations resulting from reaction of these compounds with dimethylsuberimidate (DMS)	[31]
		(ii) Longer alkyl regions in the polycation backbone increased transfection efficiency and toxicity, while increasing hydrophilicity was toxicity reducing	
		(iii) *γ*-CD polycations were shown to be less toxic than otherwise identical *β*-CD polycations	

Charge center type	(i) Amidine(ii) Quaternary ammonium	(i) Quaternary ammonium analogues exhibit lower gene expression values and similar toxicities to their amidine analogues	[30]

Termini	(i) Primary amine (ii) Histidine (iii) Imidazole	(i) Incorporation of pH-buffering moiety to polymer termini increases gene delivery, buffers acidification experienced by nanoparticles, and enhances intracellular release of nucleic acid payload	[33–35]

**Table 5:** Selected physicochemical properties of siRNA-containing, RONDEL-based nanoparticles.

Property	Method	Result
Size (diameter)	Dynamic light scattering	60 to 150 nm
Zeta potential (surface charge)	Electrophoretic mobility	0 to +30 mV
Nanoparticle molar mass	Multiangle laser light scattering (MALS)	~7e7 to ~1e9 g/mol
Stoichiometry (of 70 nm nanoparticle)	Various, including MALS	CAL101: ~10,000 molecules/particle
		AD-PEG: ~4,000 molecules/particle
		AD-PEG-Tf: ~100 molecules/particle
		siRNA: ~2,000 molecules/particle

**Table 6:** Manufacturers of primary toxicology and initial clinical lots of CALAA-01 components.

Component	Toxicology lot(s)	Initial clinical lot(s)
CAL101	Cambrex	Cambrex/Agilent Technologies
AD-PEG	Sun Bio	Sun Bio
AD-PEG-Tf	Calando Pharmaceuticals	Agilent Technologies
C05C (siRNA)	Agilent Technologies	Agilent Technologies
Fill/Finish	University of Iowa Pharmaceuticals	University of Iowa Pharmaceuticals
